# Supplementary material for: Microbiome in Male Genital Mucosa (Prepuce, Glans, and Coronal Sulcus): A Systematic Review
Source: Microorganisms. 2022 Nov 22;10(12):2312. doi: 10.3390/microorganisms10122312 (PMC9787543; doi:10.3390/microorganisms10122312)
Supplement: Supplementary file 1 [file microorganisms-10-02312-s001.zip › microorganisms-1999717-supplementary.pdf]

Microbiome in Male Genital Mucosa (Prepuce, Glans, and Coronal Sulcus): A Systematic Review

Micael F. M. Gonçalves [1,*], Ângela Rita Fernandes [1], Acácio Gonçalves Rodrigues [1,2] and Carmen Lisboa [1,2,3]

[1] Division of Microbiology, Department of Pathology, Faculty of Medicine, University of Porto, 4099-002 Porto, Portugal
[2] CINTESIS/RISE, Center for Health Technology and Services Research/Rede de Investigação em Saúde, Faculty of Medicine, University of Porto, 4099-002 Porto, Portugal
[3] Department of Dermatology and Venereology, Centro Hospitalar Universitário São João, 4200-319 Porto, Portugal
* Correspondence: mfgoncalves@med.up.pt

Supplementary File S1. Search strategy.

For MEDLINE (PubMed), Web of Science and Scopus databases:

(Microbiotas OR Microbial Community OR Community, Microbial OR Microbial Communities OR Microbial Community Composition OR Community Composition, Microbial OR Composition, Microbial Community OR Microbial Community Compositions OR Microbial Community Structure OR Community Structure, Microbial OR Microbial Community Structures OR Microbiome OR Microbiomes OR Human Microbiome OR Human Microbiomes OR Microbiome, Human) AND (Male OR Males OR Men OR Boys) AND (Genital Organs OR Genital Organ OR Organ, Genital OR Organs, Genital OR Genital System OR Genital Systems OR System, Genital OR Systems, Genital OR Genitals OR Genital OR Reproductive Organs OR Organ, Reproductive OR Organs, Reproductive OR Reproductive Organ OR Reproductive System OR Reproductive Systems OR System, Reproductive OR Systems, Reproductive OR Accessory Sex Organs OR Accessory Sex Organ OR Organ, Accessory Sex OR Organs, Accessory Sex OR Sex Organ, Accessory OR Sex Organs, Accessory) AND ((System, Urogenital OR Systems, Urogenital OR Urogenital Systems OR Genitourinary System OR Genitourinary Systems OR System, Genitourinary OR Systems, Genitourinary) OR (Membrane, Mucous OR Membranes, Mucous OR Mucous Membranes OR Mucosa OR Mucosal Tissue OR Mucosal Tissues OR Tissue, Mucosal OR Tissues, Mucosal OR Lamina Propria OR Propria, Lamina OR Muscularis Mucosae OR Mucosae, Muscularis) OR (Membrane, Mucous OR Membranes, Mucous OR Mucous Membranes OR Mucosa OR Mucosal Tissue OR Mucosal Tissues OR Tissue, Mucosal OR Tissues, Mucosal OR Lamina Propria OR Propria, Lamina OR Muscularis Mucosae OR Mucosae, Muscularis) AND (Urine)) AND (Penis OR glans OR balanopreputial sulcus OR penile coronal sulcus OR prepuce OR foreskin)

For Google Scholar database:

Microbiota AND (Male OR Men) AND Genitalia AND (Urogenital system OR Mucous membrane OR (Mucous membrane AND Urine)) AND (Penis OR glans OR balanopreputial sulcus OR penile coronal sulcus OR prepuce OR foreskin)
